# Recent Progress on Extended Wavelength and Split-Off Band Heterostructure Infrared Detectors

**DOI:** 10.3390/mi11060547

**Published:** 2020-05-28

**Authors:** Hemendra Ghimire, P. V. V. Jayaweera, Divya Somvanshi, Yanfeng Lao, A. G. Unil Perera

**Affiliations:** 1 Center for Nano-Optics (CeNo), Department of Physics and Astronomy, Georgia State University, Atlanta, GA 30033, USA; hghimire1@student.gsu.edu; 2 SPD Laboratory, Inc., Hamamatsu 432-8011, Japan; virajjayaweera@gmail.com; 3 Department of Electronics and Tele-Communication Engineering, Jadavpur University, Kolkata 700032, India; somvanshi.divya@gmail.com; 4 Hisense Photonics, Inc., 5000 Hadley Road, South Plainfield, NJ 07080, USA; ylao95@gmail.com

**Keywords:** heterostructures, split-off band, wavelength extension, device performance

## Abstract

The use of multilayer semiconductor heterojunction structures has shown promise in infrared detector applications. Several heterostructures with innovative compositional and architectural designs have been displayed on emerging infrared technologies. In this review, we aim to illustrate the principles of heterostructure detectors for infrared detection and explore the recent progress on the development of detectors with the split-off band and threshold wavelength extension mechanism. This review article includes an understanding of the compositional and the architectural design of split-off band detectors and to prepare a database of their performances for the wavelength extension mechanism. Preparing a unique database of the compositional or architectural design of structures, their performance, and penetrating the basics of infrared detection mechanisms can lead to significant improvements in the quality of research. The brief outlook of the fundamentals of the infrared detection technique with its appropriateness and limitations for better performance is also provided. The results of the long-term study presented in this review article would be of considerable assistance to those who are focused on the heterostructure infrared detector development.

## 1. Introduction

Technological advancement in material growth, processing, and characterization setups, furnished with new concepts of device structures and functions, has been widely implemented for the development of new, low-disorder, and often highly engineered material combinations, such as heterostructures [1,2]. The using layers of structurally abrupt interfaces of dissimilar compounds artificially periodic structures can be obtained [3]. Many scientists have thus used refractive indexes, bandgap, effective masses and mobilities of charge carriers, and the electron energy spectrum advantages of semiconductor heterostructures with various material combinations, architectures, and doping densities for the futuristic scientific, technical and biomedical applications [4,5,6,7,8,9,10]. The examples include GaAs/AlGaAs heterostructures [11], which have been well-studied for its potential application in high-speed digital and optoelectronic devices [12] including diode lasers [13], light-emitting diodes [14], solar cells [15] and optical detectors [8,16,17,18,19,20]. The material advantage of GaAs/AlGaAs provides excellent uniformity and large arrays. It is because there is a close match between the lattice constant of AlGaAs and GaAs. Herein, AlGaAs has a lattice constant that varies linearly between that of AlAs, 5.661 angstroms, and that of GaAs is 5.653 angstroms, depending on the mole fraction of aluminum [21]. The alternations of the aluminum fraction in the AlGaAs layer modulate the band-gap [22] and hence, one can adjust the barrier height of the designed device to match the required photon energy. Similar to GaAs/AlGaAs [23], research has been carried out on the number of other semiconductor heterostructures having various, material combinations emitter/barrier architectures and doping densities for infrared (IR) detector development [6,24,25,26,27,28].

The heterojunction detectors offer wavelength flexibility [29,30,31,32,33] and multicolor capability in these regions. The wavelength range includes short-wave infrared (SWIR) of 1–3 μm [29], mid-wave infrared (MWIR) of 3–5 μm [30], long-wave infrared (LWIR) of 5–14 μm [33], very-long-wave infrared (VLWIR) of 14–30 μm [32], far-wave infrared (FWIR) of 30–100 μm or 3–10 THz [31] and so on. Among these wavelength ranges of IR radiations, 3–5 [34] and 8–14 μm [35] are considered the atmospheric windows and are suitable for IR applications.

In this review, we stepwise describe investigations concerning the physics and applications of split-off band semiconductors heterostructures infrared detectors for the threshold wavelength extension mechanism. The description systematically explains the architectural design of the device and assesses current experimental and theoretical understanding used to improve the performance.

### 1.1. Heterostructure IR Detectors

**Figure 1:**
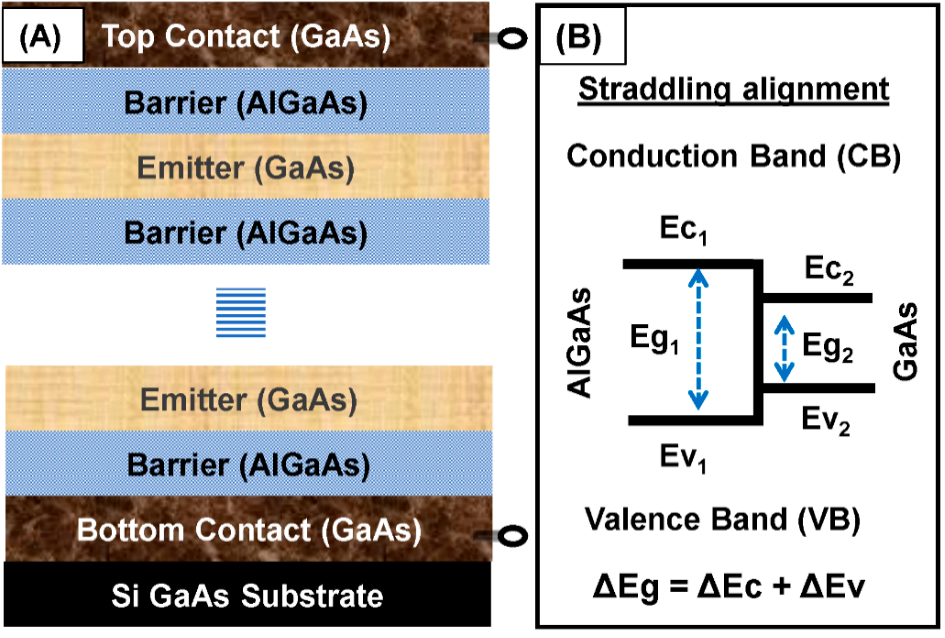
Typical architecture of a GaAs/AlGaAs heterostructure and the type depending on band alignments. (**A**) The combination of two dissimilar semiconductors in the heterostructure. The design is a p-GaAs/AlGaAs heterostructure and every layer of the emitter (p-type GaAs) is sandwiched between two layers of the barrier (AlGaAs). (**B**) Type-I heterostructure made of GaAs and AlGaAs. In such heterostructures, bandgap overlaps and Δ*E*g = Δ*E*c + Δ*E*v.

The architecture of heterostructures [36] mainly involves alternate thin layers of lattice-matched and band-gap tuned different compound semiconductors. The growth of sandwiches’ layers follows appropriate fabrication so that unique (unlike from component semiconductors) electrical and optoelectrical properties of heterostructure can be achieved. Bandgap engineered compact heterostructure is then processed with appropriate conducting rings for characterization. The processing for the fabrication also includes photolithography [37], etching to open an optical window for incidence illumination, lift-off, and others. The typical architecture of the heterostructure detector is shown in Figure 1A. In such a compact structure, each layer of the emitter is sandwiched between two layers of barriers. A sharp interface between the junctions of each layer can be achieved by making it easier for tuning the energy gap between the emitter and barrier. The development of sophisticated controllable epitaxial growth methods, such as Molecular Beam Epitaxy (MBE) and Metal-Organic Chemical Vapor Deposition (MOCVD), has allowed for the fabrication of such ideal heterojunction structures [38,39,40].

The emitter and barrier regions of heterostructure have different energy bands, and it will have an additional change in the presence of biasing [41]. In such compound heterostructures, quantum-mechanical effects such as potential discontinuity (band off-set) play a crucial role [42]. Based on the alignment of bands producing (step) discontinuity, heterostructures are classified into three types [36], type I, type II, and type III. Figure 1B shows the straddling alignment in a type-I heterostructure, where signs of the band are offset, as the two bands are opposite. In type-I heterostructures, the band gaps of one material entirely overlap with that of another, and potential discontinuities are as Δ*E*c = *E*c_1_ − *E*c_2_, Δ*E*v = *E*v_1_ − *E*v_2_ and Δ*E*g (*E*g_1_ − *E*g_2_) = Δ*E*c + Δ*E*v. Similarly, in a type-II heterostructure, *E*v_1_ > *E*v_2_ and Δ*E*c may or may not be larger than *E*g_1_. Moreover, *E*g_2_ is every so often smaller than *E*g_1_ in a type-II heterostructure. Type II staggered, (where Δ*E*c < *E*g_1_) and type II misaligned (where Δ*E*c > *E*g_1_) are two subclasses of type-II heterostructures. Type-III heterostructures are formed by combining semimetal with the inverted bands of semiconductors [43]. In this paper, we have mainly used GaAs/AlGaAs to cover type I heterostructures as shown in the above figure.

Along with technological development, there is a growing demand for advanced IR systems with better discrimination and identification. Group-IV, III-V, and II-VI semiconductor heterostructure-based [44,45] photodetectors have been studied extensively for the IR detection, from near infrared (NIR) to far infrared (FIR) region [46]. SiGe/Si heterojunctions were used to study internal photoemission LWIR detectors [33,47]. Low dimensional II-VI oxides semiconductor structures such as NiO/ZnO were also studied to understand their feasibility for light detection [48]. Similarly, studies of III-V semiconductor alloys such as InGaAs, InAsSb, InGaSb, HgZnTe, HgMnTe, GaAs, AlGaAs and their heterostructures are mainly focusing on the MWIR and LWIR detectors [49,50]. Mercury alloys led tin tellurides and selenides, and other alloy combinations were further explored for their potential as alternative material that can overcome the challenges of important semiconductor IR detector mercury cadmium telluride (HgCdTe or MCT) [27,50,51,52]. HgCdTe-based IR detectors are the most widely used for high-performance applications [50]. However, the progress has been impeded due to the fundamental material problems, such as high defect density related to growth due to the weak HgTe bond [53]. This is a serious technological problem for the mass production of HgCdTe large-sized Focal Plane Arrays (FPA), and to achieve high FPA pixel uniformity and yield.

On the other hand, Quantum Well IR Photodetectors (QWIPs), based on the GaAs/AlGaAs material system, are considered mostly for the LWIR spectral regime requiring high uniformity. The industrial infrastructure in III–V materials/device growth, processing, and packaging brought about by the utility of GaAs-based devices in the telecommunications industry gives QWIPs a potential advantage in producibility and cost [50]. However, QWIPs are not sensitive to normal incident light due to orthogonality between polarization vector of the incident photons and optical transition dipole moment [54]; therefore, light coupling structures are required for a 45° angle incidence of light which adds cost and complexity. Besides, a low operating temperature is typically required due to fundamental limitations associated with intersubband transitions [50]. Studies of Quantum Dot IR Photodetectors (QDIPs) are also making progress towards the development of IR detectors [50,55].

Because of the key pieces of evidence presented above, the studies show number of different structures and material combinations were used. The trend of exploration of new material is still increasing day by day. The superiority of heterojunction structures over the homojunction further allows for the estimation of new materials and engineering compositions in the coming days.

### 1.2. Performances of IR Detectors and Acceptable Figures of Merits

Studies have illustrated a wide range of accepted figures of merit for a meaningful comparison of the sensitivity performances of detectors [56]. These include responsivity, spectral response, specific detectivity, dark current, and the current gain. Among these performance matrices, responsivity measures the input-output gain (simply called gain) of the detector. Simply put, it is defined [57] as the ratio of the photocurrent (*I*pc) and incoming optical power (*I*o), i.e., *R* = *I*pc/*I*o = *ηqλ*/*hν* ≈ *ηλ*/1.24 (A/W), where *hν* = *hc*/*λ* is the energy of the incident photon, *h* planks constant, *c* velocity of light, the *q* charge of an electron, *λ* is the wavelength of incident light and *η* is quantum efficiency. Studies have used spectral response [58] to describe the sensitivity (ability to convert light of various wavelengths to electricity) of the photodetectors as a function of photon frequency (or wavelength). The efficiency of detection is also simply expressed in terms of quantum efficiency in various studies [59,60]. Specific detectivity [56] is another important performance matrix, which is defined as the reciprocal of noise equivalent power (NEP) normalized pre-square root of the sensor’s area (*A*) and frequency (*f*) bandwidth, i.e., $D^{*}=\frac{\sqrt{Af}}{NEP}$. Similarly, the current flowing through the detector in the absence of light, known as the dark current, is another important parameter to determine the performance of the detector [61]. The dark current characteristics are important to determine an optimum operating condition. Whilst the lowest possible dark current is desired for the operation of an IR photodetector, a certain amount of bias voltage must be applied to operate photoconductive photodetectors [61]. The study of dark current characteristics offers important insights into the device parameters, such as activation energy (Δ). Dark current in the semiconductor heterostructures is temperature dependent, which can be expressed [62,63] as:

(1) $$I_{dark}=Ae\frac{\mu F}{{\left[1+{\left(\frac{\mu F}{v_{sat}}\right)}^{2}\right]}^{1/2}}2{\left(\frac{m^{*}k_{B}T}{2\pi \hbar^{2}}\right)}^{3/2}\exp\left(-\frac{E_{act}}{k_{B}T}\right)$$

where *A* is the electrically active area of the detector, *e* is the electronic charge, *E_act_ = *Δ* − Af* − *E_f_* is the activation energy, *μ* is the mobility of the holes, *v_sat_* is the saturation velocity, m* is the effective mass, *k_B_* is Boltzmann’s constant, *T* is the temperature, *ℏ* is the reduced Planck constant, *α* is a constant parameter that determines effective barrier lowering due to the applied field *F*, and *E_f_* is the Fermi level.

### 1.3. Spin-Orbit Split-Off Band Heterostructures Infrared Detectors

The use of semiconductor heterostructures for the detection of electromagnetic (EM) spectrum blind to the human eye (UV, IR, and THz) [64] leads towards the exploration of structural concepts, their physics, theory, modeling, and experimental measurements. The study of intraband electronic transition within the valance band (heavy hole (HH), light hole (LH) and spin-orbit/split-off (SO) bands) is one of the primary characteristic features to understand the electronic process in semiconductor alloys [65,66]. Split-off band effects have been observed in the emission of GaAs metal-semiconductor field-effect transistors [67] and have enhanced the response of GaInAsP quantum wells [68]. This aspect of career transition is also implemented to develop heterostructure infrared detectors [69].

A detailed explanation of experimental SO response depending on hole transitions between LH, HH and SO bands has been presented by using GaAs/AlGaAs-based heterojunction interfacial workfunction internal photoemission (HEIWIP) detectors [70,71,72]. The active region of the basic HEIWIP detector consists of one or more periods, each consisting of a doped emitter and an undoped barrier layer. These multiple emitter/barrier layers are sandwiched between two highly doped contact layers. Depending on the doping required for ohmic contacts, the top contact may also serve as the top emitter layer. Herein, the work function (Δ) is given by Δ = Δ_d_ + Δ_x_, where Δ_d_ and Δ_x_ are the contributions from doping and the Al fraction, respectively [23]. As the Al fraction is reduced, Δ will be limited by Δ_d_, which in turn is a homojunction detector [73,74]. The detection mechanism can be divided into three main processes: (i) the photoabsorption that generates excited carriers, (ii) the escape of the carriers, and (iii) a collection of the escaped carriers.

**Figure 2:**
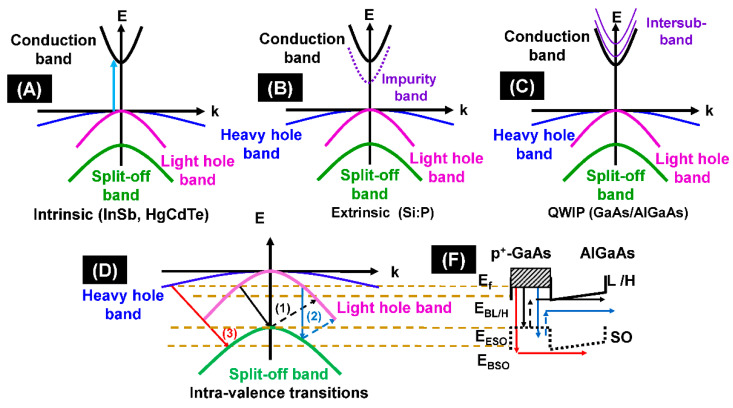
Schematic of different intervalence and intraband optical transitions showing an infrared (IR) detection mechanism. (**A**–**C**) Energy wavenumber (E-k) diagram for intrinsic, extrinsic, and quantum-well-IR photodetectors, respectively. (**D**) Split-off detector threshold mechanisms, where the IR photon excites holes from the light/heavy hole bands to the split-off band. (1) Indirect absorption followed by scattering and escape (threshold energy: *E*_ESO_ − *E*_f_). (2) Direct absorption followed by scattering and escape (threshold energy: *E*_ESO_ − *E*_f_). (3) Indirect absorption followed by escape and some scattered (threshold energy: *E*_BSO_ − *E*_f_). (**F**) Band alignment using during split-off band intra-valance transitions.

**Figure 3:**
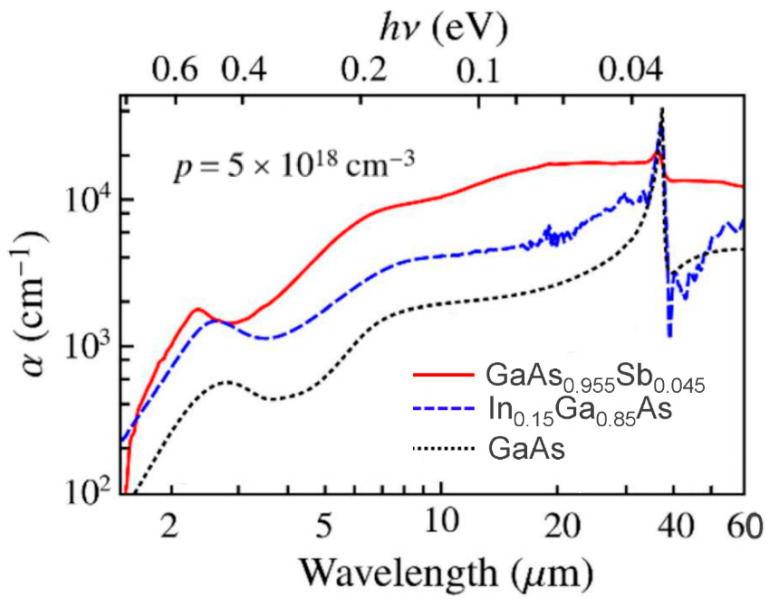
Absorption coefficient of p-type (5 × 10^18^ cm^−3^) GaAs_0.955_Sb_0.045_, In_0.15_Ga_0.85_As and GaAs as discussed [69]. The p-type GaAs_0.955_Sb_0.045_ shows the highest absorption coefficient by comparing with the other two materials.

The schematic of different intervalence and intra-band optical transitions showing the infrared detection mechanism is shown in Figure 2 in terms of energy wave vector diagrams. Figure 2A–C shows the energy wavenumber (E-k) diagram for intrinsic, extrinsic, and quantum well infrared photodetectors. Figure 2D shows Split-off detector threshold mechanisms. Infrared photon excites holes from the light/heavy hole bands to the split-off band. (1) Indirect absorption followed by scattering and escape (threshold energy: *E*_ESO_ − *E*_f_). (2) Direct absorption followed by scattering and escape (threshold energy: *E*_ESOf_ − *E*_f_). (3) Indirect absorption followed by escape and some scattered (threshold energy: *E*_BSO_ − *E*_f_). The band alignment during split-off band intra-valance transitions is shown in Figure 3F.

The spectral response of the heterojunction detector is primarily determined by the absorbing properties of the emitter, which is, in turn, determined by the electronic structure of the valence bands (VBs) and the concentration of holes. Utilizing semiconductor heterojunctions for the heterojunction detectors is subject to limitations, such as low absorption coefficient in the 3–5 μm range for p-type GaAs. The p-type semiconductor absorbs extra photons owing to optical transitions among three VBs, i.e., the LH, HH, and SO bands, contributing to an absorption band spanning primarily from 1 to 10 μm for p-type GaAs, as shown in Figure 3. The p-type semiconductors including InGaAs, GaAsSb, and GaAs indicate that the shifting of the absorption peak (between 1 and 5 μm) can be associated with the SO–HH transition. The intra-band free-carrier absorption is typically proportional to *λ*^p^, which becomes dominant in the very-long-wavelength range, where *λ* is the wavelength, and p is an exponent. p can be predicted by the Drude theory, and usually equals 1.2, 2.5, and 3.5 correspondings to scatterings by acoustic phonons, polar optical phonons, and ionized impurities, respectively. By comparing with p-type GaAs, p-type GaAsSb shows a higher absorption, which benefits a higher absorption efficiency and hence the detector performance.

Jayaweera et al. studied an uncooled infrared detector for 3–5 μm and beyond [75] by using the concept of a split-off band infrared detection mechanism [70,71]. In their study, they have experimentally demonstrated uncooled infrared detection using intra-valence bands using a set of three p-GaAs/AlGaAs heterostructure. The focus of their study is to demonstrate an uncooled infrared detection using intra-valence bands. The uncooled detection of infrared radiation is important in a wide range of applications in the civilian, industrial, medical, astronomy, and military sectors. The calculated D* value in this study is 6.8 × 10^5^ Jones at a temperature of 300 K and an SO band offset of 0.31 eV, while, for those with an SO band offset of 0.155 and 0.207 eV, D* values are 2.1 × 10^6^ and 1.8 × 10^6^ at temperatures of 140 and 190 K, respectively. It is noted that the studies on the high operating temperature that split off the transition in such a heterostructure [70,71,72] further support the infrared detection mechanism. These studies have introduced intra-valence band transitions, such as LH to heavy-hole HH transitions or HH transitions to SO band in detectors to overcome the selection rule limitations. A band diagram (E-k) of the emitter region of an S-O band detector illustrating the detection mechanism is based on the carrier transitions in the three valence bands is also demonstrated.

In the study by Lao and et al. [76], the development of structure with multi-spectral detection probability is presented. The hole transition from the HH to the LH band of p-GaAs/AlGaAs detectors show a spectral response up to 16.5 m, operating up to a temperature of 330 K where the LH-HH response is superimposed on the free-carrier response. Similarly, in another study by Matsik, and et al. [77] device modeling study of S-O band IR detector was carried out to find optimized conditions for its performance improvement. The study suggested two important device architecture modifications in order to improve device performance. One of the suggestions was to include an offset (*δE*) between the energy barriers so that the low energy barrier towards the collector side would enhance the collection. The other suggestion was to include a graded barrier on the injector side so that the carrier trapping would be reduced and the injection of the carriers to the absorber/emitter would be improved.

Altogether, the peak response due to intra-valance band transitions [70,71,78] is believed to originate from a build-up of a quasi-equilibrium Fermi level, at a fixed level, irrespective of the variation of the device parameter. Thus, the SO band was found to be the most probable energy level to build-up a quasi-equilibrium Fermi level as a consequence of the hot-phonon bottleneck effect. The study of the dark current characteristics of these IR photodetectors confirmed no compromise in the dark current due to the presence of the extended-wavelength mechanism of the photoresponse. Furthermore, the quasi-equilibrium will be built-up not only in the emitter but also in the bottom and top contact layers as long as there is photoexcitation to the SO band. The net flow of the photo-excited carriers will determine the spectral photoresponse [62,79].

In summary, HEIWIP detectors [80], based on intra-valence band transitions LH to HH transitions or HH transitions to SO band were introduced to overcome the selection rule limitations. The SO band infrared detector is a newly developed, emerging device based on the p-doped GaAs/AlGaAs system [71], which utilizes hole transition in the HH/LH band to the SO band as the detection mechanism [71]. The SO band detectors have shown promising results to be developed as an uncooled IR detector [75].

### 1.4. Effect of a Current Blocking Barrier on Heterojunction Infrared Detector

Studies have shown that incorporating current blocking architectures into detector designs alters their performances. As part of their studies, Wang and et al. [81], Rotella and et al. [82], Wang and et al. [83], Lin and et al. [84], Pal and et al. [85] and Nevou and et al. [86] have presented the use of AlGaAs current blocking layers in quantum dot IR photodetectors (QDIPs) to enhance the performance of detectors. Similarly, Stiff et al. [87], Tang [88], and Chakrabarti [89] have presented the use of a similar concept to achieve higher operating temperatures. In another study by Nguyen et al. [90], hole blocking layers have been implemented in type-II InAs/GaSb superlattice infrared photodetectors. The electron blocking and hole blocking unipolar barriers in complementary barrier infrared detectors [91] and p-type-intrinsic-n-type photodiodes [92] were also studied. The most important goal in these architectures is to increase the performance by lowering the dark current with a relatively small compromise in the photocurrent.

**Figure 4:**
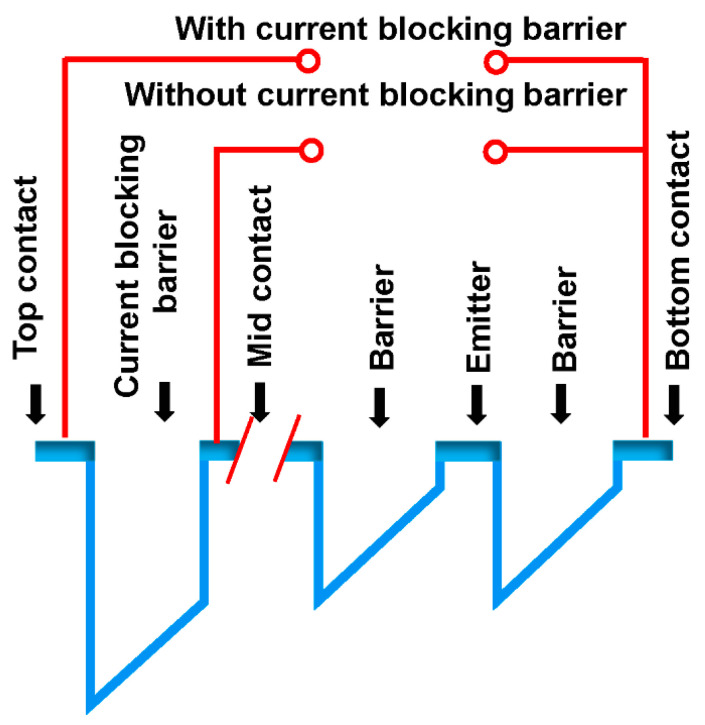
Schematic of the valence band alignment of the heterostructure under equilibrium showing the connections with and without the current blocking barrier.

Chauhan et al. have reported the performance of a p-GaAs/AlxGa1xAs heterojunction photovoltaic infrared detector, with graded barriers, operating in the 2–6 μm wavelength range [16]. They found that the incorporation of the current blocking barrier in heterostructure architecture leads to achieving a significant improvement in the specific detectivity (D*). It further reduces the dark current under photoconductive operation and increases the resistance-area product (*R*_0_*A*, *R*_0_ is the resistance at 0 V and *A* is the electrical area) under photovoltaic operation. In blocking barrier studies, measurements across the top and bottom contacts include the current blocking barrier, whilst the middle and bottom contacts measure the same sample (mesa) without the current blocking barrier, as shown in the schematic diagram of the valence band of the heterostructure in Figure 4. Herein, the implementation of a current blocking barrier increases the specific detectivity (D*) under dark conditions by two orders of magnitude to 1.9 × 10^11^ Jones, at 77 K. Furthermore, at zero bias, the resistance-area product (*R*_0_*A*) attains a five orders enhancement due to the current blocking barrier, with the responsivity reduced by only a factor of 1.5.

### 1.5. Threshold Wavelength Extension Mechanism

**Figure 5:**
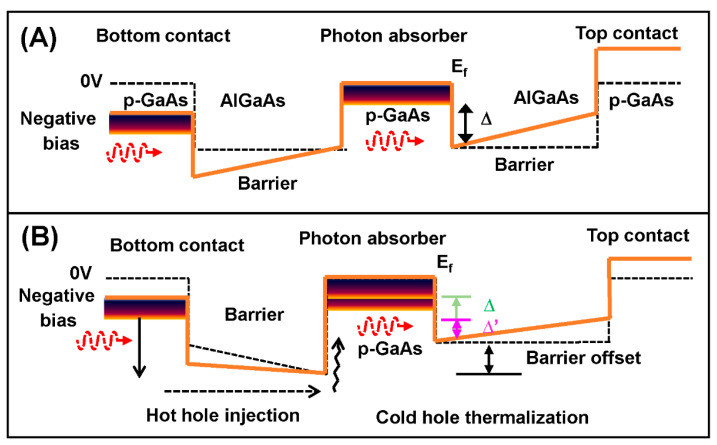
Conventional and wavelength extended photodetector. (**A**) Conventional photodetector. The threshold wavelength (*λ*_c_) of detection is determined by Δ, where *λ*_c_ = *hc*/Δ. (**B**) Wavelength extended photodetector showing barrier offset contributing to wavelength-extended photoresponse.

The study of the S-O band detector devices with the barrier offset (*δE*) and a graded barrier showed an unprecedented result in terms of the spectral range of the photoresponse: a photoresponse that is far beyond the spectral limit. In general, the standard expected threshold wavelength (*λ*_t_) of a photodetector is governed by the spectral rule ∆ = 1.24/*λ*_t_, where ∆ controls the dark current. The extended wavelength IR photodetectors are a novel class of photodetectors showing spectral photoresponse far beyond the conventional limit of Δ = *hc*/*λ*_t_. In extended wavelength detectors, the effective response threshold wavelength (*λ*_eff_) is governed by a ∆′ = 1.24/*λ*_eff_, with an effective activation energy ∆′ ≪ ∆. The valance band diagram used to demonstrate the extended wavelength photoresponse utilized an offset (*δE*) between the energy barriers in the heterostructure is shown in Figure 5B. A reference photodetector without the offset and cannot show the extended wavelength mechanism is also shown in Figure 5A.

The mechanism responsible for the extension of threshold wavelength in heterostructure detectors has been analyzed by Somvanshi et al. [93], which is based on the hot carrier effects in the semiconductor heterostructures [94,95,96]. The hot carrier effect is principally governed by the carrier–carrier and carrier–phonon scattering processes and has been widely studied in the past [96,97,98]. In general, hot carriers are created in energy states above the band edge, interact with lattice vibrations, and cold carries through carrier–carrier interactions. This leads to quasi-equilibrium Fermi distributions at a temperature much higher than the lattice temperature [97,98]. It is observed that, upon the incidence of light from an external optical source, hot holes have created in the bottom contact, photon absorber, and top contact of heterostructure detectors, as shown in Figure 5. In heterostructure detectors with band offset (Figure 5B), a net flow of hot holes has observed towards the top contact, owing to the difference in barrier heights, Δ*Ev*. However, no such flow of carriers observed in heterostructure detectors without band offset, i.e., conventional detectors (Figure 5A). The dynamics of hot hols and cold holes interaction in the p-GaAs photon absorber can be explained based on hot carrier effects. Upon the interaction of carriers in the photon absorber, the exchange of energy takes place through hole–hole and hole–phonon scattering that leads to the formation of a quasi-Fermi distribution (*E*_quasiF_) at a hot hole temperature (*T*_H_) that is significantly greater than the lattice temperature (*T*_L_). The distribution of hot holes at this quasi-Fermi level will lead to an escape of hot holes across the collector barrier when a long-wavelength photon is absorbed and the extension in threshold wavelength is observed.

Chauhan et al. have studied the photoresponse of extended wavelength IR photodetectors [99] under different bias conditions. It is observed that, with the increase in bias voltages, the photoresponse becomes stronger; however, the spectral threshold remains relatively constant. The dark current and photo-response characteristics of extended wavelength infrared photodetectors [62] studied by Chauhan and et al. show that the measured dark current of extended wavelength detectors was found to agree well with fits obtained from a 3D carrier drift model using the designed value of Δ. In contrast, the spectral photoresponse showed extended wavelength thresholds corresponding to Δ′. Since the dark current in the extended-wavelength IR photodetectors is still limited by Δ as in the conventional photodetectors, the extended wavelength mechanism offers a new avenue for the design and development of the IR photodetectors.

**Figure 6:**
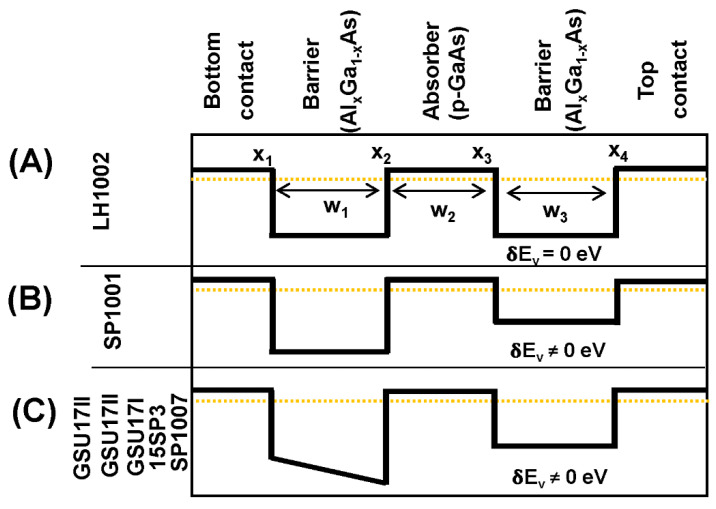
Schematic diagrams of the valence band alignment of the detector designs under equilibrium. (**A**) Detectors without barrier offset LH1002. (**B**) SP1001 consists of an emitter at the bottom, the barrier at the top, with an energy offset (*δE*v) of ~0.10 eV between the barriers. (**C**) In SP1007, 15SP3, GSU17I, GSU17II, and GSU17III have graded bottom by tuning the Al mole fraction and barrier offset δEv not equal to zero.

To understand the role of energy offset for threshold wavelength extension, a few heterostructure semiconductors were comparatively analyzed using references [100,101,102]. Schematic diagrams of the valence band alignment of the detector designs under equilibrium are shown in Figure 6. Detectors without barrier offset, LH1002 is shown in Figure 6A. SP1001 (Figure 6B) consists of a p-GaAs emitter (80 nm), an 80 nm Al_0.75_Ga_0.25_As barrier at the bottom, and a 400 nm Al_0.57_Ga_0.43_As barrier at the top. These layers are sandwiched between highly doped p-GaAs top and bottom contact layers. SP1001 has an energy offset (δE) of ~0.10 eV between the barriers. In SP1007, 15SP3, GSU17I, GSU17II, and GSU17III, as shown in Figure 6C, the bottom AlxGa1-xAs barrier (80 nm) has a graded potential profile obtained by increasing the Al mole fraction from *X*_1_ at the bottom of this layer to *X*_2_ at the top. The top AlxGa1-xAs barrier (400 nm) has a constant barrier potential profile with *X*_3_ = *X*_4_. The emitters (80 nm) are thick enough to have a bulk-like distribution of energy states. Changing the *X*_3_ causes the *δE* variation and changing *X*_1_ causes the gradient variation, as summarized in Table 1.

In summary, these studies and the devices LH1002, SP1001, SP1007, 15SP3, GSU17I, GSU17II, and GSU17III were grown on semi-insulating GaAs substrate by molecular beam epitaxy. Each heterostructure device consists of an AlxGa1-xAs barrier at the bottom, followed by a p-GaAs emitter, and then another AlxGa1-xAs barrier at the top. The bottom AlxGa1-xAs barrier is graded by increasing the Al mole fraction from a lower value x1 at the bottom of this layer to higher value *X*_2_ at the top, except for SP1001 with *X*_1_ = *X*_2_ to form a constant barrier. These emitter/barrier layers are sandwiched between the bottom and top contact layers of p-type doped GaAs. The emitter and the top and bottom contact layers are degenerately p-type doped at 1 × 10^19^ cm^−3^, whilst the AlxGa1-xAs barriers are undoped. The thickness of the p-GaAs emitter in all devices is sufficiently large to have a bulk-like distribution of energy states. 15SP3, GSU17I, and GSU17II constitute another set with varying values of the gradient ~20.6, 28.9, and 37.1 kV/cm, given by (Δ*E*v(*x*_2_) − Δ*E*v(*x*_1_))/*w*_1_ for *x*_1_ = 0.45, 0.33, and 0.21, respectively. The gradients are the only differences in these three devices. It should be noted that the device 15SP3 is common in both sets with an *δE* ~0.19 eV and gradient 20.6 kV/cm. The energy values (ΔTDIPS) corresponding to the determined wavelength threshold are also shown in Table 1. Herein, nearly doubling the barrier gradient from 20.6 to 37.1 kV/cm did not show a significant change in the wavelength threshold. These results confirm that the extended wavelength mechanism originating from the quasi-equilibrium Fermi level at a fixed energy level.

The role of barrier parameters is very critical in determining the photoresponse of extended wavelength detectors. In this view, the effect of *δE*_V_ and gradient on the extended threshold wavelength of infrared photodetectors for the temperature range up to 50 K was studied by Somvanshi et al. [103]. In this study, it is observed the *δE*_V_ is critical to obtain extended wavelength response *λ*_eff_ (≫ *λ*_t_) in IR detectors at 5.3 K; however, the gradient is needed to obtain *λ*_eff_ at 50 K. A conventional detector shows *λ*_t_ − *λ*_eff_ over operating temperature from 5.3 K and 50 K whereas the flat injector barrier and barrier-energy-offset-detector (SP1001) shows *λ*_eff_ ~36 μm at 5.3 K and 4.1 μm at 50 K. When the injector barrier changes from flat (SP1001) to graded (SP1007), the *λ*_eff_ increases from 4.1 to 8.1 μm at 50 K for a given graded injector barrier, as *δE*_V_ increases from 0.10 to 0.19 eV, the *λ*_eff_ also increases from 8.9 to 13.7 μm. The results of this study clearly indicate that by the optimization of *δE*_V_ and the gradient, infrared detectors with *λ*_eff_ (≫ *λ*_t_) can be designed to operate over a wide range of temperature.

For practical applications, to provide an advantage over the conventional detector, an extended wavelength detector should have a lower dark current and specific detectivity (D*) that is comparable or better than the conventional detector. In general, by lowering the operating temperature, the dark current can be reduced; however, this makes detectors’ operation more costly. Therefore, lowering the dark current without cooling will be an advantage, especially for longer wavelength detectors for next-generation optoelectronic devices. In extended wavelength detectors, the dark is determined by designed Δ as in the conventional photodetectors. This leads to an advantage in specific detectivity (D*) for extended wavelength detectors even though their responsivity is much lower as compared to conventional detectors. Using this idea, standard threshold semiconductor detectors could be designed to operate as long-wavelength detectors with a higher value of detectivity and reduced dark current (corresponding to the original short-wavelength threshold).

## 2. Conclusions

Objects having temperatures higher than absolute zero (*T* > 0 K) radiates the energy in the form of electromagnetic waves and so are the sources of infrared. Thus, infrared detectors can have diverse civilian and military activities applications. Major applications include: estimating heat losses in buildings, roads or any heat-emitting objects of engineering use; imaging and quality control check in biomedical use; security applications for firefighters, night vision, airports; technological applications for electrical circuit manufacturing or identifying faulty connections; application for astronomical studies and much more.

To date, many materials, and physics characteristics have been investigated in IR detection [60,104]. The example includes [105] thermoelectric power (thermocouples) [106], change in electrical conductivity (bolometers) [107], gas expansion (Golay cell) [108], pyroelectricity (pyroelectric detectors) [109], photon drag, Josephson effect (Josephson junctions) [110], internal emission (PtSi Schottky barriers) [111], fundamental absorption (intrinsic photodetectors) [112], impurity absorption (extrinsic photodetectors), low-dimensional solids (superlattice (SL) and quantum well (QW) detectors) [113], and so on. Progress in semiconductor infrared detector technology has been made in IR detector technology to improve their performance. Furthermore, studies are going on for the improvement of detector performances. In reference to this, potential applications of a heterostructure IR detector working within a wide range of temperatures, including a room-temperature environment, can be anticipated. Of course, innovation in the heterostructure architecture of thoroughly documented semiconductor compounds will create another degree of freedom in recent technology.

This study documented the present studies on the S-O band energy, and H-H/L-H transitions give rise to the photoresponse in heterostructure infrared detectors with a peak in the 3–5 μm regime, with the most important band being an atmospheric window. The incorporation of current blocking barriers to lower the dark current density and to increase the performance of heterostructure semiconductor detectors is also discussed. The importance of the wavelength threshold extension mechanism is also clearly illustrated, with its application for future IR photodetector design and development. Finally, a considerable achievement in the practical application of a new kind (unlike the conventional) of IR photodetectors, with the wavelength threshold determined in the near future, can be anticipated.

Photovoltaic infrared sensor arrays for the 3–5 μm and 8–12 μm atmospheric windows can be fabricated using similar techniques. GaAs/AlGaAs heterostructures can overcome the serious technological problem for the mass production of HgCdTe large-sized Focal Plane Arrays (FPA) [114], and to achieve high FPA pixel uniformity and yield. Large line or area arrays of such photovoltaic infrared detectors can be desired for thermal imaging and spectroscopic applications.

## Figures and Tables

**Table 1:** Summary of results from the temperature-dependent internal photoemission spectroscopy (TDIPS) fitting method with corresponding device parameters (threshold wavelength is also shown).

Device	Δ (eV)	*δE* (eV)	*A*_l_ Fraction			Thickness (nm)			Gradient (kV/cm)	Δ_TDIPS_ (eV)	*λ*t (μm)
			*x*_1_	*x*_2_	*x*_3_ = *x*_4_	*W*e	*W*_1_	*W*_2_	[Δ*Ev*(*x*_2_) − Δ*Ev*(*x*_1_)]/*w*_1_		
LH1002	0.3	0	0.57	0.57	0.57	20	20	60	0	0.2781 ± 0.0006	4.50 ± 0.01
SP1001	0.4	0.1	0.75	0.75	0.57	80	80	400	0	0.0248 ± 0.0001	50.0 ± 0.3
SP1007	0.4	0.1	0.45	0.75	0.57	80	80	400	20.6	0.0223 ± 0.000	56.0 ± 0.5
15SP3	0.4	0.19	0.45	0.75	0.39	80	80	400	20.6	0.0207 ± 0.0001	60.0 ± 0.3
GSU17I	0.4	0.23	0.45	0.75	0.3	80	80	400	20.6	0.0203 ± 0.0003	61.0 ± 0.8
GSU17II	0.4	0.19	0.33	0.75	0.39	80	80	400	28.9	0.0217 ± 0.0001	57.0 ± 0.3
GSU17III	0.4	0.19	0.21	0.75	0.39	80	80	400	37.1	0.0214 ± 0.0001	58.0 ± 0.3
